# Bone Mineralization in Electrospun-Based Bone Tissue Engineering

**DOI:** 10.3390/polym14102123

**Published:** 2022-05-23

**Authors:** Dong-Jin Lim

**Affiliations:** Department of Otolaryngology Head & Neck Surgery, University of Alabama at Birmingham, Birmingham, AL 35294-0012, USA; daniel.djlim@gmail.com

**Keywords:** bone mineralization, electrospinning, simulated body fluid, bone tissue engineering

## Abstract

Increasing the demand for bone substitutes in the management of bone fractures, including osteoporotic fractures, makes bone tissue engineering (BTE) an ideal strategy for solving the constant shortage of bone grafts. Electrospun-based scaffolds have gained popularity in BTE because of their unique features, such as high porosity, a large surface-area-to-volume ratio, and their structural similarity to the native bone extracellular matrix (ECM). To imitate native bone mineralization through which bone minerals are deposited onto the bone matrix, a simple but robust post-treatment using a simulated body fluid (SBF) has been employed, thereby improving the osteogenic potential of these synthetic bone grafts. This study highlights recent electrospinning technologies that are helpful in creating more bone-like scaffolds, and addresses the progress of SBF development. Biomineralized electrospun bone scaffolds are also reviewed, based on the importance of bone mineralization in bone regeneration. This review summarizes the potential of SBF treatments for conferring the biphasic features of native bone ECM architectures onto electrospun-based bone scaffolds.

## 1. Introduction

Bone grafting has been a standard therapeutic option for bone defects and fractures [1]. Not only do bone fractures result from accidental causes, but osteoporotic fractures are incrementally increasing in aged populations worldwide [2]. Osteoporosis is a skeletal disorder that gradually leads to fragile fractures when a failure-inducing force (e.g., trauma) is applied, and it is a general term describing weakened bone density and bone quality [3]. The imbalance of osteoblastic bone resorption and osteoblastic bone formation results in osteoporosis. Several factors for osteoporosis have been identified, such as genetic, intrinsic, exogenous, and lifestyle factors [4]. Moreover, osteoporosis-related fractures occur in various anatomical positions in the body, such as the spine, hip, and wrist [5]. The annual cost of osteoporotic fractures and osteoporosis was $16 billion per year in the United States [6]. Almost 10 million people older than 50 years old were reported to have osteoporosis, and 1.5 million were estimated to be subject to fragility fractures in the United States [7]. Additionally, osteoporosis has been a leading disease of mortality worldwide, due to increasing life expectancy and longevity [8]. In an ideal case, autologous bone grafting and self-transplantation in the same patient would be considered for treating the bone fractures [9]. However, a limited supply of grafts and patient compliance while harvesting osseous matter has deterred patients from self-transplantation [10]. Alternatively, allopathic bone grafts can be collected from human cadavers [11]. Surgeons have often performed allografts, but the potential risk of contamination, including donor-derived infections, is a deterrence from using these therapeutically viable scaffolds [12,13]. Similar to other tissue-derived products, allografts often hold unexpected pathogenic infections such as bacteria, viruses, and prions [14]. More importantly, allogeneic bones often exert cellular and humoral immune reactions [15]. There are also other donor-based grafts, but they are from nonhuman species. Although such grafts can solve the shortage issue, they need to be prepared through more thorough sterilization protocols, reducing their original potential in osteoinductive properties [16]. Based on a recent study showing the clinical outcomes of bone grafts using allografts, xenografts, and alloplastics in sinus lift or ridge preservation procedures, it has easily been confirmed that an allograft has a better capacity for the creation of new bones than xenografts [17]. As a different approach for bone grafts, demineralized bone matrix (DBM) has been utilized [18,19]. DBM is prepared through a complex process where collected bones are soaked or washed in strong acid reagents (e.g., hydrochloric acid or nitric acid) to eliminate potential contamination and the risk of disease transmission [20,21]. However, the osteoinductivity of DBM is dependent on variations in bone quality from individual donors as well as batch-to-batch process variations [22,23]. In a practical setting, the variation of bone-forming potential in different commercially available DMB products has been documented. It is thought that this is due to the inconsistency of the manufacturing process [24]. The first and foremost benefit of BTE is the provision of well-designed osteoinductive and/or osteoconductive scaffolds for the improvement of bone density and bone quality [25]. Osteoinductive scaffolds are capable of permitting the growth of bone cells. In contrast, osteoconductive scaffolds represent the ability to stimulate primitive and undifferentiated cells (e.g., mesenchymal stem cells [26] and induced pluripotent stem cells [27]) towards bone-forming cells [28]. As a promising scaffolding platform, electrospun-based materials display interconnected porous structures, and become either cellular-based or drug-based scaffolds, thereby increasing the intrinsic potential of bone regeneration, as well as conferring an extrinsically regenerative potential for bone regeneration [29]. For example, a study created a multilayered synthetic fibrous scaffold comprising β-tricalcium phosphate (TCPs) and poly(ε-caprolactone) (PCL) electrospun nanofibers to form bone-like ECMs by the osteoconductive TCPs and the biocompatible elastic PCL nanofibers [30]. Using goat-derived bone marrow stromal cells (BMSCs), the authors proved that electrospun composite scaffolds could increase the osteogenic differentiation of exogenously supplied BMSCs. In this regard, BTE outperforms conventional bone allografts, which are immunogenic and often limited, due to supply shortages [31]. An engineered bone scaffold should have a functional resemblance to a natural ECM, with osteoconductivity for better regenerative outcomes [32]. Electrospun bone scaffolds have highly porous interconnected structures, thereby maximizing their surface area [33]. Moreover, electrospun nanofibers can quickly become a temporary bone substitute by conferring reinforced mechanical strength onto as-spun nanofibers via numerous cross-linking strategies [34]. Hence, the nanofibrous appearance of the as-spun electrospun bone scaffolds dictate the potential for electrospun-based scaffolds in recapitulating the native bone ECM environment, which is one of the critical engineering parameters of BTE [35].

To create functionally augmented electrospun-based scaffolds in BTE, the most common approach is to mineralize electrospun scaffolds to enhance osteogenic potential by mimicking the native bone ECM microenvironment, leading to successful bone grafts and repairs. Incredibly, simulated body fluid (SBF) is a robust but straightforward recipe for inducing hydroxyapatite (HA) and apatite-based inorganic clusters onto the surface of the electrospun scaffolds (Table 1) [36]. These inorganic solutions enable us to create functional electrospun bone scaffolds that are capable of inducing bone regeneration via a dynamic interaction between the synthetic grafts and the endogenously or exogenously provided cells that are responsible for bone regeneration. This study aims to outline numerous electrospinning technologies that are employed in electrospun-based bone scaffolds, and to describe the science of SBF development. Lastly, examples of biomineralized electrospun scaffolds are explored, in order to understand and to expand the potential of biomimetic scaffolds in BTE.

## 2. Bone: Dynamic and Biphasic Tissue

**Figure 1:**
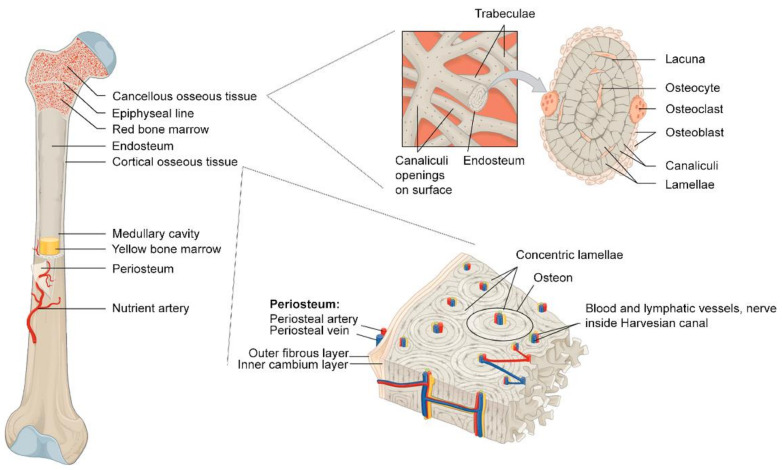
Schematic image of bone anatomy. Reprinted with permission from Ref. [47]. Copyright 2017 MDPI. More details on “Copyright and Licensing” are available via the following link: https://www.mdpi.com/ethics#10, accessed on 15 April 2022.

Bone is a vital tissue that is responsible for essential functions in the body: the mechanical support of the body, locomotion, and dynamic reservoir units for biological components and blood cells (Figure 1) [43]. Bone provides minerals such as calcium, magnesium, and phosphate, and holds bone marrow, particularly red bone marrow, occupied inside the bone tissue to mature and to distribute blood cells. Rather than being a supportive physical frame, bone is a dynamic organ where bone cells and hematopoietic stem cells (HSCs) play a role in maintaining whole-body homeostasis. There are four types of cells in the bone: bone-forming osteoblasts, bone-resorbing osteoclasts, and bone-embedded osteocytes, which are known as a modulator of the cellular activities of osteoblasts and osteoclasts in the dynamic process of bone regeneration [44,45]. The last type of cell, the bone lining cell, has a relatively unclear role or mechanism for coupling bone resorption to bone formation [46]. To fulfill the bone’s role in the body, bone is made of two different types of matter, organic and inorganic components.

### 2.1. Microstructural Bone Formation: Biphasic Aspects of Bone

The organic part of the bone is typically composed of type I collagen and other structural proteins. Type I collagen (Col1) represents nearly 90% of this part, and contributes to bone strength [48,49]. With a few exceptions, Col1 is a triple-helix of three chains: two α1 chains and one α2 chain [50]. Col1 is enzymatically converted from secreted type I procollagen, like other collagens [51]. With regard to the structural aspect, Col1 is one of the fibrillar collagens that is characterized by a triple-helix conformation and repeated (Gly-X-Y)_n_ sequences [52]. Gly is glycine, while X and Y are different amino acids, meaning that theoretically, more than 400 combinations are possible. However, the Gly-Pro-Hyp triplets are the most prominent combinations present, increasing the molecular stability and the natural intermolecular actions. Collagen molecules can spontaneously form collagen fibrils, which then become collagen fibers and bundles [53]. Such collagen fibrils are also spontaneously created from purified collagen in aqueous solutions [54]. Based on the authors’ observations, collagen liquid crystal can be induced at an acidic pH, since the positively charged residues of collagen help to maintain a liquid state without aggregations, while the rising pH with the aid of ammonia vapors gradually decreases the net charge of the collagen monomers, leading to the formation of collagen fibrils. The brief mechanism of type I collagen fibrillogenesis is mentioned below. Due to the long helical domain of each chain in Col1, the newly-formed three chains spontaneously assemble into triple-helix type I procollagen in the collagen synthesizing cells [55]. In type I procollagen, N-terminal and C-terminal propeptides prevent the formation of the premature collagen fibril, and modulate the fibril assembly [56]. The N- and C-telopeptides are typically 16 and 25 amino acids long, affecting the final self-assembly structures of Col1 [57]. For example, a partial loss of those peptides results in the poor self-assembly of Col1. In contrast, the loss of each telopeptide forms different kinds of self-assembled collagen, indicating that there are other kinetic mechanisms of collagen fibrils. When the type I procollagen is secreted into the extracellular space, abundant proteolytic enzymes such as matrix metalloproteinases (MMPs) and bone morphogenetic protein 1 (BMP-1) are responsible for initiating spontaneous collagen fibril formation [58]. Such enzymes target the N-terminal and C-terminal propeptides from the type I procollagen. For example, a disintegrin and a metalloprotease with thrombospondin type I motifs (ADAMTS), ADAMTS-2, can cleave the N-terminal propetides [59]. Likewise, ADAMTS-14 is also observed to have similar aminoprocollagen peptidase activity [60]. Bone morphogenetic protein-1 (BMP-1) can also excise the C-terminal propetides [61].

The inorganic bone minerals represent approximately 60% of bone tissue by weight and 40% by volume [62]. The bone minerals have two distinct roles: (1) they act as a reservoir of ions for the body, and (2) they are embedded in the organic components of the bone to create a light and tough natural composite material [63]. Bone minerals are important in ion homeostasis, regulating approximately 99% of the calcium and 85% of the phosphorus in the body [64,65].

Similarly, sodium and magnesium in the bone account for at least half of the required levels in the body (nearly 90% and 50%, respectively) [66]. In nature, both components become a biological composite with multi-level hierarchical properties [67]. Mineralized collagen can be considered to be a building block that creates the hierarchical structure of bone. The mineralized collagen by itself is thought to be a reinforced collagen composite where thin calcium phosphate-based crystals are intercalated between collagen nanofibrils [68]. Mineralized collagen fibrils eventually become a unit of lamellar bone structures. In this biphasic bone structure, the collagen-based organic ECM regulates the cellular activities of the bone-resident and the bone-forming cells. At the same time, the HA-based ECM plays a role in the structural support of bone [69]. As a result, a bundle of fibrillar collagens can be observed. Interestingly, each fibrillar collagen can undergo a further enzymatic cross-linking process, leading to a lysine-mediated intermolecularly cross-linked collagen bundle [70]. From the viewpoint of the structural locations of the cross-linking, this occurs between the short non-helical peptides (N- and C-terminal telopeptides) and a helical portion of an adjacent collagen molecule [71]. It is known that all major collagens, types I, II, and III, have four cross-linking sites at equivalent locations of each collagen molecule. Moreover, Col1 has unique cross-linking products, called pyridinoline cross-links, which interconnect between the N-telopeptide and the helix intermolecular cross-linking domain of the Col1 molecules [72]. Hence, biomineralization and extra cross-linking properties contribute to the complex hierarchy of the bone.

### 2.2. Macrostructural Bone Formation: Vascularization and Ossifications

In a macroscopic aspect, bone is a highly vascularized connective tissue, where bone vasculature participates in bone development (endochondral and intramembranous ossification), bone remodeling, and the regeneration of bone [73]. The trabecular bone is spongy bone tissue that is observed at the ends of a long bone, while compact bone, also called cortical bone, is the dense exterior bone [74]. The intricate vascular network pervading the Haversian and Volkmann’s canals is observed in the cortical bone. The Haversian canals are the longitudinal route of blood vessels in the cortical bone, while the Volkmann canals interconnect the blood vessels of the Haversian canals [73]. Haversian systems or osteons are the basic units of compact bone. Each osteon is composed of lamellae of compact bone tissue derived from mineralized collagen fibrils and osteocytes that are founded in small cavities of each osteon, called lacunae [75]. Thin tubes called canaliculi from each lacuna serve not only as paths for blood supply, but also for the spaces that allow osteocytes to connect to each other through gap junctions [76]. Notably, the center of each osteon is called the Haversian canal, which contains blood vessels and nerve fibers that are parallel to the long axis of the bone. Bone can form via intramembranous ossification (osteogenesis) and endochondral ossification [77]. Endochondral ossification is the process of bone formation, during which chondroblasts (mesenchymal progenitor cells for cartilage formation) form a membrane called the perichondrium around a cartilage template. These chondroblasts become chondrocytes that secrete growth factors for recruiting blood vessels towards the perichondrium. Then, the perichondrium becomes the bone-forming periosteum. In contrast, intramembranous ossification is the immediate bone-forming process without the involvement of a cartilage model, which is shown in endochondral ossification. Endochondral ossification can be found in the long bones, whereas most skull bones are formed through intramembranous ossification [78,79].

### 2.3. Bone Remodeling and Bone Healing

Bone remodeling is the two-step process by which osteoclasts break old bone tissues down, followed by bone deposition, which replaces new bone tissues through the cellular activities of bone-forming osteoblasts [79]. As a dynamic tissue of the body, the bones are constantly under bone remodeling for the following reasons: (1) remodeled bones support newly applied mechanical stresses upon the bone architecture; (2) bone remodeling maintains ion homeostasis by regulating calcium and phosphate ions in the body; (3) bone remodeling repairs microdamage to the bone [80].

In fracture healing, there are several types of fractures after post-reduction treatment; the broken bones undergo the healing process. Damaged blood vessels that are associated with the fractures create a hematoma (localized bleeding) and induce clot formation around the damaged bone [81]. The clots help to recruit new blood vessels and become fibrous granulation tissues called soft calluses. Approximately 1 week after injury, the soft callus turns into a fibrocartilaginous callus, which eventually becomes a bony callus approximately 2 months later. Further bone remodeling and reshaping occur over several months to complete the stages of the fracture repairs [82].

## 3. Electrospinning Technologies: Electrospun Scaffolds in Bone Mineralization

**Figure 2:**
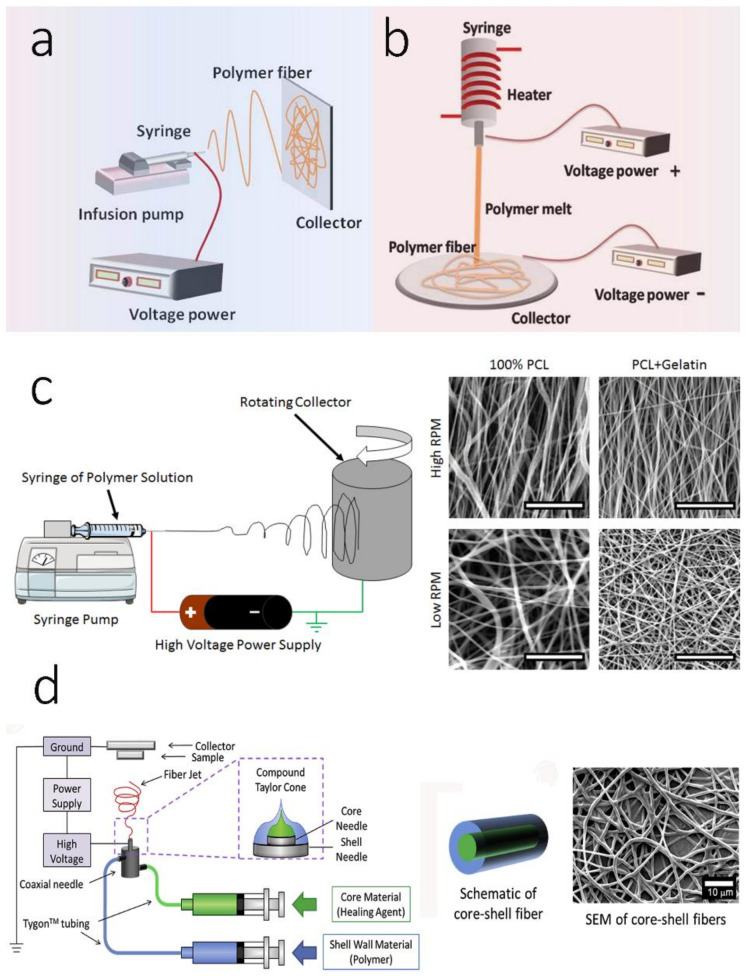
Schematic images of different electrospun fabrication techniques: (**a**) monoaxial electrospinning; (**b**) melt electrospinning; (**c**) aligned electrospinning; (**d**) coaxial electrospinning. (**a**,**b**) reproduced with permission from Ref. [89]. Copyright 2017 Elsevier; (**c**) reproduced with permission from Ref. [90]. Copyright 2016 PLOS under a Creative Commons Attribution 4.0 International License; (**d**) reproduced with permission from Ref. [91]. Copyright 2016 Elsevier. More details on “Copyright and Licensing” are available via the following link: https://www.mdpi.com/ethics#10, accessed on 15 April 2022.

Although there are many technical approaches for creating nano-sized ECM-like threads or fibers such as self-assembly, phase separation, and electrospinning, the versatile electrospinning strategy outweighs other methods in terms of material selection, post-modifications, and adaptability to other scaffold platforms (Figure 2) [83,84,85]. Different versions of the original electrospinning strategy enable us to confer more delicate morphological features onto the final electrospun nanofibers for regenerative bone scaffolds (Table 2). For example, a coaxial electrospinning technique creates dual growth-factor-loaded electrospun scaffolds to enhance osteoconduction and osteoinduction [86]. Likewise, triaxial electrospun scaffolds have the potential for developing multifunctional nanofibers. A recent study showed that a tripolymeric triaxial electrospun scaffold supports the cellular activity of rat adipose-derived stem cells (ADSCs) [87]. In the melt electrospinning technique, distinctive non-woven nanofibrous architectures can be fabricated by maintaining a polymeric solution in a highly viscous liquid while performing the electrospinning [88].

### 3.1. Monoaxicial Electrospinning

William Gilbert initiated the basic concept of electrospinning in 1600. Under an electric field, he discovered the cone-shaped water droplet, which was eventually named the ‘Taylor cone’, since Geoffrey Taylor documented the mathematical modeling of the conical shape of a polymer solution when applying a strong electric field, in his seminal works in the 1960s [100]. Using a simple instrument, a sufficient electric potential can be imposed upon a polymer solution to create the Taylor cone. The basic setup of an electrospinning machine has four components: (1) a high-voltage power supply, (2) a syringe pump, (3) a spinneret, and (4) a conductive collector. Before electrification, a driving force that is applied to a polymer solution in a syringe creates a pendant-shaped droplet in the tip of a spinneret, where surface tension governs and results in the spherical shape of the solution. However, when an electric potential is accumulated in the solution, the electrostatic force sufficiently surpasses the surface tension to create a Taylor cone, continuously drawing polymeric fibers onto a conductive collector. During the charged polymeric liquid’s travel, it converts into a series of solid nano-sized threads due to solvent evaporation. Electrospun scaffolds fabricated by a traditional electrospinning technique hold several favorable features for BTE. They have a large surface area-to-volume ratio, a high porosity, and a similar morphological shape to native bone ECM [101]. Before using the electrospun scaffold in BTE, biocompatible polymeric foams were investigated, giving an insight into the preferred material design parameters for BTE. Using poly(α-hydroxy acid) foam scaffolds, a previous study demonstrated that a preferable bone scaffold would have an interconnected internal structure with at least 90% porosity and a 100 to 350 µm pore size [102]. It is not convenient to fabricate such highly porous micro-channeled structures using the phase separation technique, but the electrospinning technique can easily make bone remodeling scaffolds with minimum effort. A simple but meaningful study showing the potential of the electrospinning technique in BTE has been reported [103]. In this study, the authors created a microporous and non-woven PCL scaffold by a monoaxial electrospinning technique, demonstrating the attachment and growth of mesenchymal stem cells (MSCs) derived from the bone marrow of neonatal rats. Because of this simple and efficient manufacturing method, monoaxial electrospinning remains a standard technique for creating a regenerative bone scaffold. A growth factor, basic fibroblast growth factor (bFGF), for supporting BMSCs was successfully incorporated into a monoaxial electrospun scaffold. It showed a sustained release of bFGF over time [104]. Rabbit BMSC seeded onto monaxial poly(lactic-co-glycolic acid) (PLGA) was expanded and stimulated by slowly released bFGF to produce type I collagen, as well as fibronectin, over 1 week. In general, monoaxial electrospinning is an essential and effective tool for creating functional electrospun scaffolds for BTE.

### 3.2. Melt Electrospinning

The melt electrospinning technique can create a straight and stable polymer jet. Instead of using a solvent-based polymer solution for electrospinning, a heating element around a syringe allows us to make a molten polymer with relatively high viscosity and low conductivity. An ejected stable jet of molten polymer from an electrospinning apparatus, in general, creates thicker nanofibers but well-designed electrospun architectures over two or three dimensions. Due to its lack of requirement for solvents, melting electrospinning is also considered to be a green nanotechnology. Because solvent residuals in a biomaterial-based product affect the biocompatibility of the product, electrospun scaffolds obtained using this novel technique would be a promising approach for creating more sophisticated but also safer electrospun-based bone scaffolds [105]. A study utilized melt electrospinning to create a hybrid scaffold using conventional electrospinning and melt electrospinning [106]. Using silk fibroin (SF) and PCL, the authors successfully fabricated SF/PCL nano/microfibrous composite scaffolds and proved that the composites were supportive of the osteogenic potential of human mesenchymal stem cells (hMSCs) isolated from the alveolar bones of patients during oral surgery. Similar to the advancement of different electrospinning technologies, melt electrospinning has also evolved into melt electrowriting (MEW), a combined technology of electrospinning and fused deposition modeling (FDM) that is the most well-known extrusion-based additive manufacturing method [107]. One of the fabrication benefits of MEW is that it can create a highly porous structure with adjustable filament size (5 to 50 µm) [108]. A study created a flexible and osteoconductive fibrous composite made of PCL and HA based on the MEW process [109]. This study incorporated HA nanoparticles into a PCL solution to create a composite solution followed by the melt electrospinning writing process. The fabricated HA/PCL composite had a high degree of porosity (96−98%) and fully interconnected pore architectures, thereby supporting the osteoactivity of human osteoblast cells.

### 3.3. Aligned/Oriented Electrospinning

Recent progress in BTE has also encouraged electrospinning technology to fabricate more aligned and ordered nanofibers that assist with the design of osteoinductive and osteoconductive electrospun scaffolds. Compared to non-aligned nanofibers, aligned nanofibers modulate cell adhesion and migration, and they affect the production of ECM and cytokines [110]. NIH-3T3 fibroblast cells were attached and spread along with the aligned nanofibers by modifying the cellular cytoskeleton onto the aligned electrospun nanofibers. The topological characteristics of a substrate can influence cellular behaviors, including growth and differentiation [111]. In BTE, recent studies using aligned electrospun nanofibers have demonstrated the role of fiber orientation to the extent to which several stem cells undergo osteogenic differentiation. In a study, random and parallel poly (l-lactic acid) (PLLA) nanofibers were fabricated to evaluate the effects of fiber orientation on cell morphology, proliferation, and the differentiation of osteoblast-like MG63 cells [112]. MG63 cells grew along the aligned direction of the PLLA electrospun nanofibers. However, no statistically conclusive data showed better osteogenic potential in the aligned nanofibers. In contrast with the above study, using an osteoblast-like cell line, a study used human bone marrow mesenchymal stem cells (hBMSCs). It demonstrated the positive effects of both the aligned PLLA electrospun nanofibers and the aligned microfibers on the osteogenic differentiation of the stem cells [95]. The aligned nanofibers (AN) had average fiber diameters of 580 ± 10 nm, whereas the aligned microfibers (AM) in this study were demonstrated on a micro-size scale (1.21 ± 0.15 μm). Both the aligned or random electrospun nanofibers enhanced the osteogenic potential of the hBMSCs. Compared to the random electrospun fibers (random nanofiber; RN and random microfiber; RM), both the aligned fibers caused the hBMSCs to extend along the elongated direction of the fibers. In addition, hBMSCs on the aligned fibers showed faster migration speeds than the random fibers. Lastly, such morphological behaviors of hBMSCs on the aligned fibers reflect improvements in osteogenic differentiation, which were assessed via alkaline phosphatase (ALP) staining and alizarin red (ARS) staining. ALP is one of the most reliable markers produced from osteogenic cells, while ARS staining is a common assay technique for the cellular mineralization of various osteogenic cells [113,114]. Based on the hBMSCs’ cellular behaviors as measured on days 7 and 10, the AN group showed stronger ALP staining intensity than did other groups (AM, RN, and RM). Similarly, ARS staining performed 21 days after seeding the stem cells on each substrate indicated that the AN group exhibited a significantly higher staining intensity than the other groups. These findings likely confirm that the aligned nanofibers are better osteoinductive bone scaffolds than the normal electrospun scaffolds that are usually fabricated using a conventional electrospinning apparatus. To create aligned nanofibers, numerous invented approaches have been addressed. A straightforward means of collecting aligned fibers is to use a rotating collector. Instead of using a static and flat collector, a study adopted a mandrel collector rotating at high speeds (e.g., 4500 rpm) while collecting nanofibers that were continuously ejected from a Taylor cone [115]. Further improvements in fabricating aligned nanofibers were also made by applying auxiliary counter electrodes onto the surface of a mandrel. The auxiliary electrodes created a converged electric field, thereby forming an aligned and dense electrospun scaffold without any apparent change in the average diameters of the aligned nanofibers [116]. As a different technique for alignment, a study used two separated parallel conductive collectors onto which the charged nanofibers were stretched and covered between the gap between the collectors [117]. The concept of the conductive parallel collector was also adopted in a mandrel [118]. In this technique, evenly spaced copper wires aligned through the barrel of a mandrel were placed to create a circular drum that served as a collector of the electrospun nanofibers. Because of the combinational effect of the mechanical stretching force by a mandrel and the electrostatic interactions between a parallel collector, aligned nanofiber sheets can be collected easily without disturbing the aligned structure. A different study introduced a new rotor-type collector with perpendicularly standing fins to assist wind-electrospun filaments in large amounts during electrospinning [119]. Whereas the above technologies have been focused on improving or modifying some of the four components in an electrospinning machine, magnetic electrospinning (MES) uses a polymer blend containing a small amount of magnetic materials to magnetize the ejected polymer, thereby creating aligned nanofibers under a magnetic field [120]. Additional forces, such as the post-drawing force and the centrifugal force, are also utilized to create aligned nanofibers. A study using PLLA nanofibers already aligned by approximately 60% confirmed that applying a post-drawing force in an oven at a high temperature (110 °C) using a manual drawing device results in the improved alignment of the electrospun PLLA nanofibers by up to 90% [119]. Using an additional centrifugal force in electrospinning, large-scale aligned nanofibers can be obtained as an electrospun mat with a rapid fabrication time [121]. Notably, the aligned electrospun nanofibers affect the morphological features of the attached cells. The aligned poly(d,l-lactic acid) (PLA) nanofibers changed the cellular morphology of the bone marrow stromal cells and showed an increased degree of calcium deposition during osteogenic differentiation [122]. An inverse relationship between alignment and osteogenic potential has recently been documented [123]. Human embryonic stem cell-derived mesenchymal progenitor cells (hES-MPs) on random gelatin-coated PCL electrospun nanofibers showed better rates of mineralization and osteogenic differentiation, as confirmed by both the ALP and ARS activities. Inversely, the mature osteoblast cell line MLO-A5 showed enhanced ALP activity and more calcium deposition in the same but aligned scaffold. The dependency of both the cell-specific and the nanofiber alignments on developing electrospun bone scaffolds should be considered as a design parameter for the pursuit of an ideal electrospun-based scaffold for BTE.

### 3.4. Multi-Axial Electrospinning

Compared to monoaxial electrospinning, multi-axial electrospinning requires a special spinneret and greater consideration when choosing appropriate experimental parameters to succeed in fabricating multi-layered electrospun nanofibers. However, the fabricated multi-layered electrospun nanofibers would expand the potential for electrospun scaffolds in multiple biomedical applications, including BTE. The most frequently studied multi-axial electrospinning technique is coaxial electrospinning, which is similar to monoaxial electrospinning, except for a special spinneret and an associated modification (two pump-driven reservoirs). In terms of a spinneret in the coaxial electrospinning technique, a coaxial spinneret is an outer-shell spinneret that is concentrically aligned. For co-axial electrospinning, several experimental considerations have been addressed: (1) a sheath solution is usually being with higher viscosity and better conductivity compared to a core solution; (2) the flow rate of a sheath solution has to be faster than that of a core solution; (3) there is low interfacial tension present between the core and sheath solutions used; and (4) relatively volatile solvents are recommended for a sheath solution to create a stable Taylor cone formation [124]. In addition, the selection of solvents for multiple-axial electrospinning is also a cumbersome parameter for generating successful core/sheath nanofibers [125]. According to the author’s comments, each solvent system for both the core and sheath polymeric solutions affects the final structure of the core/sheath nanofibers. When the core and sheath solvents are miscible, each polymer used should not be soluble in another solvent system. Otherwise, the solutes could precipitate, even within the tip of the spinneret used. In contrast, immiscible solvent systems create stable core/sheath nanofibers, even if each polymer can diffuse into another solvent system, while miscible solvent systems are suitable for creating nanofibers through coaxial electrospinning. In addition to the advantage of monoaxial electrospun nanofibers, the coaxial nanofibers bring more versatile features to the final tissue-engineered products [126]. The features can be summarized as follows: (1) the sheath portion can serve as a biophysical protective barrier for deposited drugs within the core portion; (2) the release of the drugs can be modulated by controlling the thickness of each portion while electrospinning; and (3) the mechanical properties of the coaxial nanofibers are adjustable to meet the mechanical requirement of the native bone tissues. Due to the morphological benefits of the coaxially electrospun scaffolds, for example, numerous studies in BTE have been found elsewhere. Using a rotating needle collector, a study fabricated a coaxial PCL/HA-added PLA electrospun tube that was capable of growing human mesenchymal stem cells [127]. Additionally, BMP-2 growth factors were slowly released from the fabricated tubes, irrespective of the presence of HA. A study used tussah silk fibroin to incorporate HA into the sheath of coaxial electrospun scaffolds [128]. This coaxial electrospun scaffold also used tussah silk fibroin for the core. The core/sheath nanofiber can also be used to deliver a drug that has been deposited within the core of the core/sheath nanofibers. A study incorporated TCP nanoparticles into the core of the core/sheath electrospun scaffolds and compared the release profile of the TCP nanoparticles from the coaxial PLA nanofibers with morphologically different PLA nanofibers, including monoaxial nanofibers [129]. When the TCP nanoparticles were added, the average size of the electrospun nanofibers was significantly changed. The monoaxial nanofibers had average fiber diameters of 450 ± 72 nm, whereas the coaxial nanofibers had approximately double diameters (890 ± 125 nm). Owing to the successful embedding of the TCP nanoparticles into the core part, the coaxial nanofibers had a smooth surface, indicating that no TCP nanoparticles were found on the surface of the nanofibers. As expected, the release of the TCP nanoparticles from the coaxial electrospun were markedly delayed and extended for 36 days. While the monoaxial electrospun released most TCP nanoparticles within several days, the coaxial electrospun maintained constant release profiles. Multi-axial electrospinning would be an excellent platform that delivers agents (e.g., BMP-2 or -7 growth factors) for promoting bone regeneration. In addition to serving as a biomimetic bone-like ECM, the core-sheath nanofibers can provide therapeutic agents in the desired manner. Moreover, the structural advantage of the core-sheath nanofibers is that these distinctive layers could retain the therapeutic activity of the incorporated agents Hence, the multi-axial electrospinning strategy makes electrospun scaffolds capable of satisfying the unmet needs of various bone defects.

## 4. Simulated Body Fluid for Bone Scaffold Mineralization

Mineralized collagen fibrils are responsible for the elastic modulus of bone and bone fracture toughness. In addition to the density of apatite, the deposited HA size, orientation, and localization are parameters that affect the strength of bone [130]. An SBF is a solution preparation that creates the bone-like apatite layer upon various substrates, including polymers, ceramics, and metals [131]. Inspired by human blood plasma, several SBFs have been formulated by modifying different ion compositions (Table 3). By convention, a conventional SBF is called c-SBF. Its compositional formulation is similar to human blood plasma, with the exception of two ions, chloride anion (Cl^−^) and bicarbonate anion (${\mathrm{HCO}}_{3}^{-}$) [132]. The concentration of Cl^−^ in c-SBF was higher than that of blood plasma, whereas c-SBF has significantly lower ${\mathrm{HCO}}_{3}^{-}$ levels than in blood plasma. An interesting study recently showed that regular cell culture media might be an alternative to normal SBF [133]. Since the inception of an SBF formation, a series of studies has been performed to improve the efficacy of SBF formulations. Compared to the conventional SBF (c-SBF) formulation, for example, three improved formulations have been studied: (1) Revised SBF (r-SBF) represents a reduction in Cl^−^ and an increase in HCO_3_^−^ concentration compared to those of c-SBF; (2) ionized SBF (i-SBF) has the lowered concentrations of two divalent cations (Ca^2+^ and Mg^2+^) compared to those of r-SBF; and (3) modified SBF (m-SBF) has a moderate HCO_3_^−^ level compared to the levels of both r-SBF and i-SBF [134]. By comparing three formations, m-SBF was selected as a suggested SBF formulation, since m-SBF makes bone-like apatite onto substrates and demonstrates a similar degree of storage stability to c-SBF when stored at 36.5 °C for 7 days. Revised SBF (r-SBF) and modified SBF (m-SBF) were much closer to the compositions of human blood plasma, but those formulations were lacking in creating bone-like apatite for calcium-based materials. A revised SBF formulation called *n*-SBF, which stands for a newly improved SBF, was also studied [135]. For example, a study created a microporous composite scaffold in which natural gellan gum (GG) and nanoparticulate bioactive glass (BAG) were blended in a solution containing calcium chloride (CaC1_2_) and mineralized with flow SBF [136]. In this study, a perfused SBF flows in the axial direction with or without the presence of vertical direct compression to create the best biomimetic scaffold containing HA for BTE. The authors observed that the perfused SBF flow forms cauliflower-like HA within the GG–BAG scaffolds that are comparable to HA crystals observed in vivo. However, the application of direct compression reduces the formation of HA, followed by the destruction of the GG–BAG scaffolds.

## 5. Simulated Body Fluids for Electrospun-Based Bone Scaffolds

**Figure 3:**
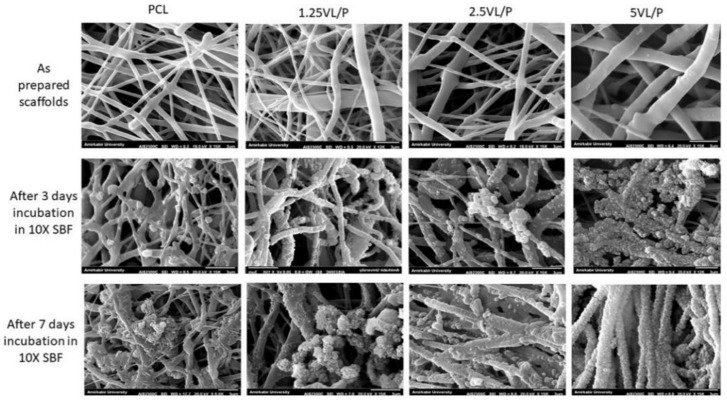
Scanning electron microscope images of as-prepared scaffolds and after 3 and 7 days incubation in 10× SBF. The formation of spherical apatite-like crystals increased significantly after adding nanohybrids to the scaffolds. For the legends, pure PCL and VD3·LDH/PCL electrospun scaffolds containing 1.25, 2.5, and 5 wt% of vitamin D3 are presented as PCL, 1.25VL/P, 2.5VL/P, and 5VL/P, respectively. Reprinted with permission from Ref. [139]. Copyright 2020 Elsevier. More details on “Copyright and Licensing” are available via the following link: https://www.mdpi.com/ethics#10, accessed on 15 April 2022.

Mineralized electrospun scaffolds have been fabricated by the simple immersion of electrospun nanofibers in different SBFs, ranging from regular SBFs to concentrated SBFs (Table 4). In this section, various mineralized electrospun scaffolds have been addressed to confirm the potential of this excellent but straightforward strategy for electrospun biomineralization. PCL electrospun nanofibers were pretreated with NaOH (2N, 24 °C for 12 h) and mineralized in SBF for up to 21 days [137]. From the analysis of the selected area using electron diffraction (SAED) pattern and energy dispersive spectroscopy (EDS) measurements, it was confirmed that the mineralized PCL nanofibers exhibited a similar ring-shaped pattern to that of crystalline apatite. The ratio of Ca/P was approximately 1.71, which was comparable to the value of HA (Ca/P~1.67). When used to cultivate MC3T3-E1 subclone 4 cells, which are known to be a good model of in vitro osteoblast differentiation via ECM signaling, the HA-mineralized PCL nanofibers showed better osteogenic performance potential than in the case of the non-treated nanofibers [138]. In another study using PCL electrospun scaffolds, vitamin D3 (VD3) containing anionic SDS micelles (SDS; sodium dodecyl sulfate) were intercalated between layered double hydroxides (LDHs), which are known to be the most biocompatible inorganic nanocarriers in the drug delivery system [139]. Then, the fabricated VD3·LDH/PCL electrospun scaffolds were mineralized within concentrated SBF (10×) (Figure 3). Interestingly, an increase of the VD3·LDH nanohybrids within the PCL-based electrospun scaffolds enhanced apatite-like crystal formation in vitro. Based on recent findings regarding vitamin D3 (VD3) in inducing osteoblastic differentiation, this study incorporated VD3 and evaluated the osteogenic potential of the VD3 loading electrospun bone scaffolds with human osteoblast-like MG-63 cells.

For improving the mineralization of PLLA electrospun nanofibers, a concentrated SBF (×10) was utilized after treatment with either NaOH (0.1 M) or co-blending with gelatin (10%) [140]. Such pretreatments assist with the mineralization of the electrospun nanofibers within 2 h, in the concentrated SBF. However, co-blending with gelatin was better at yielding the stress and elastic modulus than the NaOH-treated PLLA nanofibers, indicating the gelatin-derived hydrophilic properties of the 10% gelatin/PLLA nanofibers could facilitate mineralization. In another study, amorphous calcium particles (ACPs) were added to enhance the mineralization process onto the PLA electrospun nanofibers, which are similar to another PLLA hydrophobic synthetic polymer [141]. Only a 1-day treatment in an SBF solution showed significant growth of inorganic HAs on the surface of ACP containing PLA nanofibers. In contrast, no change was observed on the surface of the pure PLA nanofibers, even after immersion in SBF for 7 days.

**Table 4:** Exemplary uses of simulated body fluid (SBFs) in electrospun-based bone scaffolds.

Type of Electrospun Scaffold	Treated SBF Protocol	Descriptions	Ref.
PLGA/collagen/gelatin (2:1:1 weight ratio)	10× m-SBF	The mineralized PCG nanofibers were fragmented and loaded with BMP-2 mimicry peptides ^1^ for alveolar bone regeneration in vivo.	[142]
Liginin/PCL	1.5× SBF	The fibrous liginin/PCL films were completely coated by HA within 5 days.	[143]
Alginate/PLA	1.5× SBF	The alginate/PLA composite was crosslinked by Ca^2^^+^ and mineralized. Anionic alginate assists with the nucleation and growth of calcium phosphate apatites.	[144]
Polysilsesquioxane (POSS)-loaded PLA	1× SBF	The POSS-PLA showed acceleration in HA mineralization.	[145]

^1^ Bone morphogenetic protein-2 (BMP-2) is a well-known growth factor capable of inducing osteogenesis. The BMP-2 mimicry peptides are derived from BMP-2, and they have a poly-glutamic acid residue (E7 Tag) for electrostatic interaction between the peptides and HAs.

Likewise, carbonate nano-hydroxyapatite (*n*-HA) was incorporated into an electrospinning solution to induce mineralization [146]. Although electrospun nanofibers fabricated from a copolymer of L-lactide and DL-lactide (PLDL) were not mineralized properly for a 7-day immersion within a 1.5-fold SBF solution, *n*-HA/PLDL nanofibers have successfully undergone the process of full HA mineralization after 3 days of immersion. Similarly, PCL nanofibers containing HA nanoparticles (NPs) were mineralized within an SBF solution for 10 days at 37 °C [147]. It was confirmed that the embedding of HA-NPs initiates the crystallization of HA from the SBF treatment, and that an incremental addition of HA-NPs in PCL colloidal solutions improved the formation of bone-like apatite.

**Figure 4:**
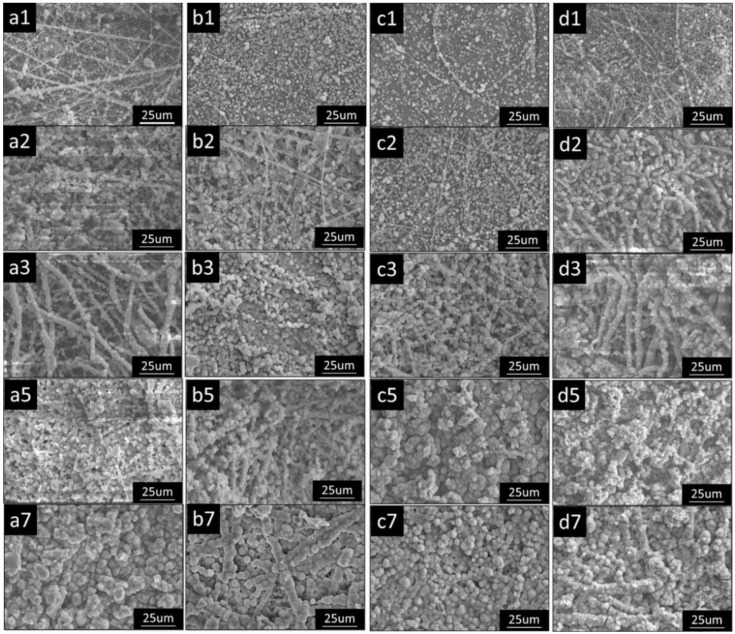
Scanning electron microscope images of the gradual deposition of minerals onto PLLA/gelatin composite nanofibers over time in different concentrated SBF (2.5×) fortified with amino acids (2.5 mM) at 37 ± 0.2 °C. The numbers following the alphabets a–d indicate the soaking time (days). (**a1**–**a3**,**a5**,**a7**) 2.5 SBF-blank; (**b1**–**b3**,**b5**,**b7**) 2.5 SBF-Gly; (**c1**–**c3**,**c5**,**c7**) 2.5 SBF-Arg; (**d1**–**d3**,**d5**,**d7**) 2.5 SBF-Asp. The number shown in each panel of the figure represents the days of each SBF treatment. Magnification of 1000×. Reprinted with permission from Ref. [148]. Copyright 2015 Elsevier. More details on “Copyright and Licensing” are available via the following link: https://www.mdpi.com/ethics#10, accessed on 15 April 2022.

For better mineralization, a study used gelatin and amino acids (e.g., glycine, aspartic acid, and arginine) [148]. A polymeric blend of PLLA and gelatin (1:1 weight ratio) was used for electrospinning, while each amino acid (2.5 mM) was supplemented into a concentrated SBF (2.5×). At different incubation periods, the authors observed significant differences in mineralization. Compared to concentrated SBF (2.5×), the presence of amino acid facilitates HA crystal formation, transforming it from amorphous calcium phosphate to hierarchical HA (Figure 4). Among the amino acids, the authors also noted that glycine had promoted the formation of well-evolved needle-like HA crystals. It was speculated that adding amino acids into SBF would assist with inducing biomineralization for electrospun-based bone scaffolds.

Interestingly, a study used a charged protein that could enhance the process of mineralization while applying a concentrated SBF (10×) [149]. In this study, phosvitin (PV), one of the egg (usually hen eggs) yolk phosphoproteins, was utilized to obtain a better rate of mineralization on the surface of the collagen nanofibers [150]. The involvement of PV in the concentrated SBF (×10) resulted in the rapid formation of apatite within 4 h, which was comparable to HA, and confirmed by EDS analysis. Instead of incorporating an additive or a treatment with NaOH for modifying the surface of the pure electrospun nanofibers, a study employed a collagen coating technique onto the prepared PLGA nanofibrous mesh (NFM) [151]. After collagen coating, a concentrated SBF (5×) treatment resulted in the formation of tiny HA nanoparticles onto NFM. The HA-coated NFM supported the growth of the MC3T3-E1 osteoblasts and their subsequent differentiation. Additionally, the osteogenic differentiation of the BMSCs proved the potential of the HA-deposited collagen-coated PLGA electrospun mesh in BTE. Onto the blend of electrospun nanofibers from hydroxyethylcellulose (HEC) and polyvinyl alcohol (PVA), concentrated SBF (10×) was also utilized to coat them with bone-like apatite within 2 days [152].

**Figure 5:**
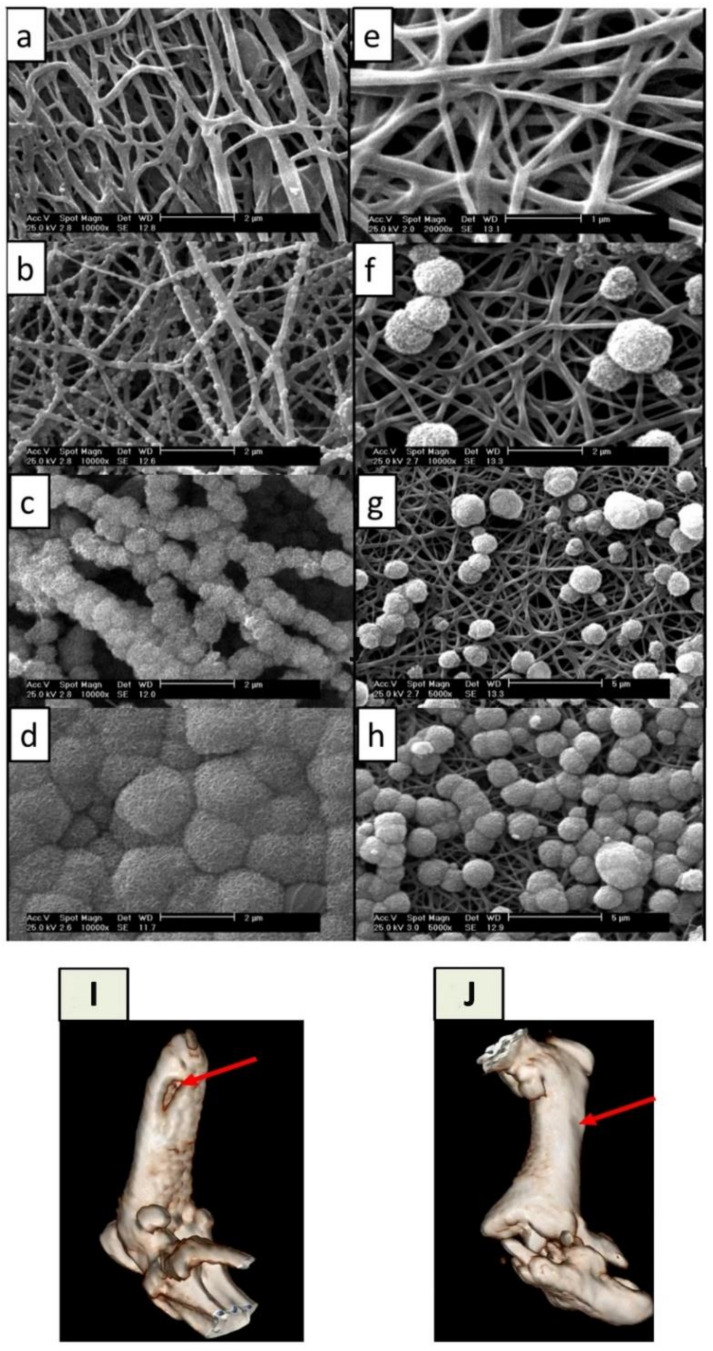
Scanning electron microscope (SEM) images of pristine carbon nanofibers (P-CNFs) and pre-treated carbon nanofibers (T-CNFs) incubated in a normal SBF. (**a**) T-CNFs-12 h, (**b**) T-CNFs-24 h, (**c**) T-CNFs-48 h, (**d**) T-CNFs-72 h, (**e**) P-CNFs-12 h, (**f**) P-CNFs-24, (**g**) P-CNFs-48 h, and (**h**) P-CNFs-72 h. (**i**,**j**) 3D computed tomography (CT) imaging of in vivo repair of a defective femur via mineralized carbon nanofibers (M-CNFs). Diagnostic 3D imaging (CT scan) of femur bone defects after 8 weeks of injury. The arrow shows the unrepaired defective site in the control group (**i**) and the bone defect repaired by normal tissue growth caused by the M-CNFs (**j**). Reprinted with permission from Ref. [156]. Copyright 2020 Nature publishing group. More details on “Copyright and Licensing” are available via the following link: https://www.mdpi.com/ethics#10, accessed on 15 April 2022.

The mineralization technique is also a promising strategy for electro-conductive biomaterials that are capable of transferring electric stimulations onto cells. The electrical stimulation would help to promote bone regeneration. Under periodic electrical stimulation (1 h per day, 0.4 ms pulse, and 20 Hz frequency) with different voltages (1, 5, 10, and 15 V), human fetal osteoblastic cells (hFOB 1.19) cultured on the surface of anodized nanotubular titanium were responsive [153]. Significantly, an electric field generated at 15 V enhanced osteoblast growth up to 72% after 5 days. In addition, osteoblasts move toward the cathode, and osteoclasts move in the opposite direction (the anode) [154]. As mentioned in this study, this cell galvanotaxis is a unique property that may contribute to the development of new BTE for difficult-to-access bone fractures. Electro-conductive electrospun carbon nanofibers (CNFs) would be an excellent substrate for electrical stimulation in BTE applications [155]. Using an SBF formulation, a study successfully synthesized HA-mineralized electrospun CNFs derived from polyacrylonitrile (PAN) [156]. This study confirmed that a typical SBF post-treatment created hydrophilic and biomimetic CNFs while preserving the conductive properties of carbon. Even after 24 h, SBF post-treatment resulted in the uniform mineralization of CNFs when pristine CNFs (P-CNF) were pretreated with a concentrated NaOH solution (5 M) at 45 °C. The pre-treated CNFs (T-CNFs) were thought to form mineral phase nucleation sites on the surface of the T-CNFs due to the exposure of the surface carbonyl groups. In a rat bone defect model where 6 mm segmental damage was created in the femurs of Wistar rats, the mineralized CNFs (M-CNF) completely restored the bone defects of the femurs within 8 weeks (Figure 5). In summary, the SBF treatment is a standardized protocol for conferring biomineralization onto electrospun nanofibers. Interestingly, there have been attempts to boost the biomineralization process via the following approaches: (1) pretreating as-spun scaffolds to create a more wettable charged surface; (2) adding functional agents that induce or expedite SBF nucleation; (3) coating the surfaces of electrospun scaffolds to enhance the initiation of the mineralization process.

## 6. Conclusions

Improvements in the development of synthetic bone grafts have aspired to fill the gap between the constant need for bone substitutes and the shortage of bone tissues for bone defects and bone fractures, including osteoporotic fractures. Electrospinning has been extensively explored as a promising manufacturing strategy for creating a biomimetic bone scaffold. Electrospun scaffolds have high porosity, a large surface-area-to-volume ratio, and structural similarities to native bone ECM. For bone-like scaffolds, the imitation of the biphasic nature of native bone ECM is critical because mineralization makes the bone harder and more capable of mechanically supporting the body, implementing the ability to move, and supplying a dynamic reservoir of biologically essential elements (e.g., minerals, blood cells, and growth factors). Therefore, an in-situ mineralization technique with different simulated body fluids (SBFs) has been extensively adopted to create fully biomimetic electrospun-based bone scaffolds. This reliable and straightforward post-treatment has successfully fabricated various mineralized electrospun bone-like scaffolds. As reviewed in this study, recent research progress in SBF-based mineralization protocols has made mineralized electrospun scaffolds much more versatile in repairing bone defects and fractures.

## Figures and Tables

**Table 1:** Recent examples of using simulated body fluids (SBFs) in different applications ^1^.

Name	Fold-Change ^2^	Descriptions	Purpose	Implications
${\mathrm{HCO}}_{3}^{-}$ modified SBF [37]	1-fold	${\mathrm{HCO}}_{3}^{-}$ ions were incrementally supplemented. (5, 10, 15, 20, and 27 mM)	Addition of ${\mathrm{HCO}}_{3}^{-}$ ions affected the composition and structure of formed calcium phosphates.	Under conditions lower than 20 mM, only B-type carbonated apatite precipitated, while 27 mM ${\mathrm{HCO}}_{3}^{-}$ resulted in the formation of A-type carbonated apatite as well.
Selenate added 1.5× SBF [38]	1.5-fold	0.15 mM SeO_4_^2−^ ion was added, and ion concentration was increased to 1.5×. Subtractions: None	Incorporating Se into the bone-like apatite structure to obtain a coating with potential anti-cancer and anti-bacterial properties on the surface of Ti6Al4V.	Adding 0.15 mM selenate ion did not yield secondary calcium phosphate phases other than HA. Se was shown to inhibit the proliferation of osteosarcoma cells without affecting the proliferation of normal bone cells in vitro. The coating was also shown to inhibit the growth of *Staphylococcus epidermidis*.
Modified SBF [39]	2-fold	Concentrations of CaCl_2_ and KH_2_PO_4_ were doubled. Subtractions: None	Deposition of CaP ^4^ onto electrospun chitosan and polyvinyl alcohol (PVA) fibers	Spherical CaP crystallites (average diameter of 350 nm) with nano-sized β-TCP ^5^ crystalline plates with low crystallinity formed on the fibers starting from the first day.
Modified SBF [40]	2-fold	Concentrations of CaCl_2_ and KH_2_PO_4_ were doubled. Subtractions: None	Deposition of CaP on chitosan substrates, which were prepared by spin coating of chitosan on Ti	Mg ion-incorporated bone-like apatite was synthesized by incubating the chitosan-coated Ti in m-SBF.
10× SBF [41]	10-fold	Ion concentration was increased to 10×. Subtractions: ${\mathrm{HCO}}_{3}^{-}$ and SO_4_^2−^ ions were omitted. No buffering agent was used.	The formation of HA ^3^ onto gelatin-siloxane microspheres was fabricated via a single emulsion method in modified 10× SBF solution using microwave energy (600 W).	The homogeneity and speed of mineralization increased in 10× SBF solution with the microwave-assisted method, compared to the conventional coating systems. Biomimetic monodispersed HA exhibited nanoscale morphology and good cytocompatibility with human osteosarcoma cell lines (MG-63).
Boron added SBF (B-SBF) [42]	10-fold	5–17 mg boric acid (H_3_BO_3_) was added, and the ion concentration was increased to 10×. Subtractions: ${\mathrm{HCO}}_{3}^{-}$ and SO_4_^2−^ ions were omitted. No buffering agent was used.	Producing biomimetic boron-doped HA with the support of microwave for coating tissue scaffolds	Freeze-dried chitosan tissue scaffolds were coated with boron-doped HA via the microwave-assisted biomimetic process. No buffers were used in the preparation of 10× SBF. The addition of boron did not alter the crystallinity of HA.

^1^ This table is a revised version from the original table in Ref. [42] with permission. Copyright 2020 Elsevier. ^2^ Fold-changes were estimated based on the concentration with respect to [Ca^2+^] in the conventional SBF (c-SBF) formulation. ^3^ Hydroxyapatite (HA); ^4^ Calcium phosphate (CaP); ^5^ β-tricalcium phosphates (β-TCP).

**Table 2:** A comparison of different modes of electrospinning (ES) for bone regeneration ^1^.

ES Modes	Advantages	Limitations	Recent Examples
Monoaxial	Simple installation Easy to operate	Random patterns Lack of tensile strength	Regenerated cellulose non-woven electrospun scaffolds [92] HA-embedded poly(3-hydroxybutyric acid-co-3-hydrovaleric acid) (PHBV) random nanofibers [93]
Melt	Three-dimensional structure Larger pore size Diverse diameter range Eco-friendly method	Expensive setup Mostly amorphous fibers and thermal degradation	Multilayered PCL/ gelatin scaffolds (through both monoaxial and melt modes) [94]
Aligned	Aligned structure Guided oriented arrangement and elongation of cells Decreased size in diameter Good mechanical properties	Complex setup Clogging or jet instability	Aligned poly (L-lactic acids) (PLLA) nanofibers [95] Aligned nano-HA-incorporated poly(D,L-lactide-co-glycolide) (PLGA) electrospun scaffolds [96]
Multi-Axial	Core-shell structure Versatility and flexibility for functional scaffolds	Complex setup Difficult material selection and fabrication	Coaxial poly (3-hydroxybutyrate-co-4-hydroxybutyrate)/poly (vinyl alcohol) (P34HB/PVA) nanofibers [97] Triaxially in-situ calcium phosphate fabrication in gelatin electrospun nanofibers [98]

^1^ This table is a revised version of the original table in Ref. [99]. Copyright 2020 MDPI. More details on “Copyright and Licensing” are available via the following link: https://www.mdpi.com/ethics#10, accessed on 15 April 2022.

**Table 3:** Formulations of various simulated body fluids (SBFs).

Ions (mM)	Blood Plasma						
	Total	Dissociated	c-SBF	r-SBF	i-SBF	m-SBF	*n*-SBF
Na^+^	142.0	142.0	142.0	142.0	142.0	142.0	142.0
K^+^	5.0	5.0	5.0	5.0	5.0	5.0	5.0
Mg^2^^+^	1.5	1.0	1.5	1.5	1.0	1.5	1.5
Ca^2^^+^	2.5	1.3	2.5	2.5	1.6	2.5	2.5
Cl^−^	103.0	103.0	147.8	103.0	103.0	103.0	103.0
HCO_3_^−^	27.0	27.0	4.2	27.0	27.0	10.0	4.2
HPO_4_^2−^	1.0	1.0	1.0	1.0	1.0	1.0	1.0
SO_4_^2−^	0.5	0.5	0.5	0.5	0.5	0.5	0.5

## Data Availability

Not applicable.
